# Surface Characterisation of Atmospheric Pressure Plasma Treated Cotton Fabric—Effect of Operation Parameters

**DOI:** 10.3390/polym10030250

**Published:** 2018-02-28

**Authors:** Chi-Wai Kan, Wai-Shan Man

**Affiliations:** Institute of Textiles and Clothing, The Hong Kong Polytechnic University, Hung Hom, Kowloon, Hong Kong; wsman@yahoo.com

**Keywords:** surface, atmospheric pressure plasma, cotton, operation parameters

## Abstract

The surface of cotton fibre was modified by atmospheric pressure plasma treatment (APPT), using gas as the carrier. Effects of variations in four operational parameters, discharge power, oxygen flow rate, jet-to-substrate distance and speed of the jet movement were examined. Morphology of surface of cotton fabrics was examined by generating Scanning Electron Microscopy (SEM) images. Elementary composition of the surface of the fabric was examined by X-ray Photoelectron Spectroscopy (XPS) and Fourier Transform Infrared Spectroscopy-Attenuated. Total Internal Reflectance (FTIR-ATR) was used for examining functionality of the surface. In this study, we revealed that the operational parameters would physical and chemically after the surface characteristics of the cotton fibre. Physically, cracks and grooves were noted in the cotton fibre surface after APPT. Chemically, the oxygen content in the cotton fibre surface was increased after APPT. When the O/C ratio is taken into consideration, the surface oxidation was a steady effect in applying APPT for treating cotton fibre in this study.

## 1. Introduction

Surface characteristics of fibres and fabrics can be altered by plasma treatment and this does not much impact the bulk properties of fibres and fabrics [1,2]. Precise effect of plasma treatment, i.e., the change it induces in characteristics of surface of the fabric, depends upon which gas is used in the treatment [3,4,5]. Polymerising gases like methane, ethylene and ethanol, which have compositions with high proportions of carbon and hydrogen atoms, are quite popular and find widespread usage [5]. When gases that are non-polymerising are used for plasma treatment, modification of the surface is caused by way of cross-linking, ablation, oxidation and sometimes grafting. The differences between effects of different gases are mainly attributable to the type of chemical reactions they induce [1]. The textile industry now uses atmospheric pressure plasma treatment (APPT) quite extensively for altering surface characteristics of fabrics, one distinct advantage being that it is a continuous process [6,7,8,9,10]. 

Use of oxygen in plasma treatment is proven to be good for enhancing wettability of textile materials. Under the influence of plasma, the oxygen plasma species can be produced as follows [11]:

(i) Ion and electron formation

e^−^ + O_2_ → O_2_^+^ + 2e^−^

(ii) Atom and radical formation

e^−^ + O_2_ → O + O

and

(iii) Generation of heat and light

e^−^ + O_2_ → O_2_* + 2e^−^

O_2_* → *hv*

e^−^ + O → O*

and

O* → *hv*

From (i) to (iii), O_2_* and O* are excited states of O_2_ and O, those species formed in (i) to (iii) are in equilibrium state and they are active plasma species for the oxygen plasma treatment. In previous studies, we have shown that APPT with oxygen constitutes a beneficial pretreatment of 100% woven [12] and knit fabrics [13] for improved dyeing, particularly when the fabric is pigment dyes [14,15,16] because adhesion of pigment is improved, resulting in better depth of dyeing. APPT helps several other types of dyeing also [17].

Therefore, in this study, we study the effects of APPT on fabric surface using atmospheric pressure plasma jet (APPJ) system. In the APPJ system four operational parameters, i.e., (1) discharge power; (2) flow rate of oxygen; (3) jet moving speed; and (4) jet-to-substrate distance, would be used. The effect of these four operational parameters on the surface characteristics of cotton fabric would be evaluated instrumentally. 

## 2. Experimental Section

### 2.1. Materials

One hundred percent ready-for-dyeing cotton fabric of 249 g/m^2^ (0.52 mm thickness) was used. Before testing fabric specimens were subjected to washing with diluted acetone (99%, GR Grade) for 5 min, followed by drying in an oven at 50 °C for 10 min. After that, the specimens were conditioned at 20 ± 2 °C and 65 ± 2% relative humidity for at least 24 h before use. 

### 2.2. Atmospheric Pressure Plasma Treatment—Atmospheric Pressure Plasma Jet (APPJ)

**Figure 1:**
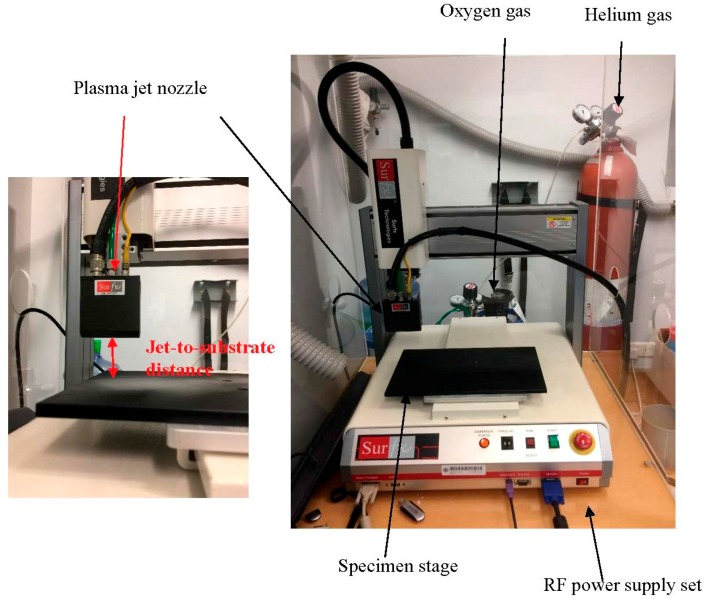
Set-up of the APPT [18].

An APPJ, Atomflo^TM^ 400 machine (Rectangular nozzle, AH-500L, Surfx Technologies LLC, Redondo Beach, CA, USA) machine was used for atmospheric pressure plasma (APP) treatment. Active area was set at 50 × 20 mm^2^. The fabric was placed vertically below the plasma jet nozzle (Figure 1). Frequency of generating plasma was set at 13.56 MHz. While oxygen (O_2_, 99.7% purity) was used as the reactive gas, Helium (He, 99.995% purity) was used as the carrier gas in the tests. Flow rate of the carrier gas (Helium) was set at 30 L/min. Details of the four operational parameters (discharge power, flow rate of oxygen, jet moving speed and jet-to-substrate distance) are shown in Table 1. After the APPT, fabric specimens were conditioned 20 ± 2 °C and 65 ± 2% relative humidity for at least 24 h prior to evaluation. 

Effects of APPT vary with operational parameters. For ascertaining impact of individual parameters, value of the specific parameter being studied was varied while keeping the remaining three parameters unchanged, i.e., if the effect of discharge power is studied, other three parameters such as flow rate of oxygen, jet moving speed and jet-to-substrate distance are kept constant.

### 2.3. Scanning Electron Microscopy (SEM)

SEM images (magnification 4000×) of surfaces of APP treated fabrics were generated by a scanning electron microscope (SEM, JSM-6490, JEOL Ltd., Tokyo, Japan), with 20 kV accelerating voltage for examining the magnitude of changes in surface topography caused by the treatment. This was at <100 nm level.

### 2.4. X-ray Photoelectron Spectroscopy (XPS)

X-ray Photoelectron Spectroscopy (XPS) was used for examining composition of the cotton fibre surface while a SKL-12 spectrometer (Leybold Heraeus-Shenyang, Shenyang, China) with a VG CLAM 4 multi-channel hemispherical analyzer (equipped with Al/Mg twin anodes) was used for examining the substrate. The X-ray Photoelectron Spectrometer was operated with non-monochromatic Mg Kα (1253.6 eV) radiation for characterizing APP treated substrate under vacuum condition (8 × 10^−8^ Pa) with applied voltage of 10 KV and working current of 10 mA. Surface contamination was removed by argon ion gun (5 KeV) at 2 × 10^−4^ Pa and 10 mA current. For analysing the spectra and obtaining elementary results (binding energy range: 0–1200 eV), we used software XPSPEAK 4.1 (Informer Technologies, Inc., Los Angeles, CA, USA). Besides, deconvolution analysis was carried out for the contents of each chemical component with C1s and two distinct sub-peaks corresponding to C–C (285.0 eV) and C–O (286.5 eV) were analysed [18,19].

### 2.5. Fourier Transform Infrared Spectroscopy-Attenuated Total Internal Reflectance (FTIR-ATR)

Surface functionality of the APP treated specimens was examined by an infrared spectrophotometer (Spectrum 100, Perkin Elmer Limited, Hong Kong, China) with the mode of Attenuated Total Internal Reflectance (ATR). This technique (FTIR-ATR) can measure chemical composition up to a depth of 500 nm in the fabric. IR frequencies of absorbance and the relative concentrations of certain functional groups are identified by the intensity of peak signals. As many as 64 scans at 4 cm^−1^ resolution were taken for each FTIR spectrum. Measurements obtained include the area of absorption peaks for C–OH stretching at 3270 cm^−1^ and C–O stretching at 1313 cm^−1^ [20,21]. 

## 3. Results and Discussion

Modification of surface is a process that accelerates as concentration of active species on the fabric surface increases progressively. Control of oxygen concentration and discharge power helps strike an equilibrium between physical etching and chemical modification and that leads to stability since it determines energy contained in the gaseous mixture [1]. The distance between the jet (nozzle) and the substrate (fabric) determines the distance the plasma mixture has to travel for reaching the fabric surface. On the other hand, duration of the treatment, i.e., the time period for which active plasma species is able to interact with the surface of the material, is determined by the speed at which the jet moves. Thus, speed of the jet and the distance between the jet and the fabric surface together determine efficiency and sufficiency of active species reaching the fabric surface and causing chemical and etching changes [10].

### 3.1. Effect of Discharge Power

**Figure 2:**
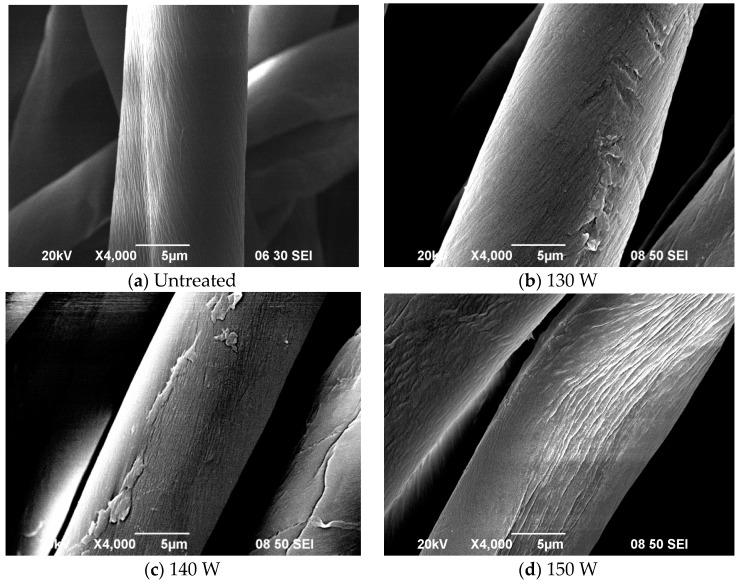

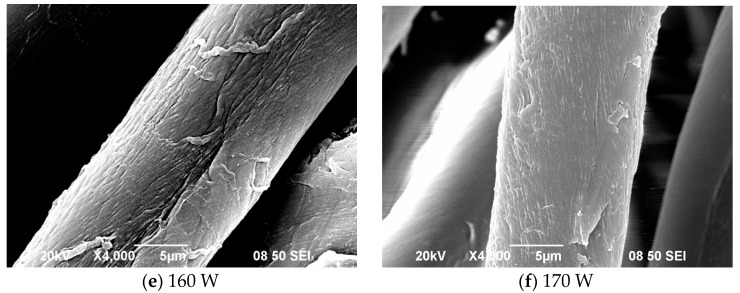
SEM images of (**a**) untreated cotton fibre (Ref. [21], with permission); APP treated cotton fibre with discharge power of (**b**) 130 W; (**c**) 140 W; (**d**) 150 W; (**e**) 160 W; and (**f**) 170 W (APP conditions: Oxygen flow rate = 0.3 L/min; jet-to-substrate distance = 3 mm and jet moving speed = 5 mm/s).

The extent to which discharge power affects the morphological change on the surface was examined by generating SEM images before and after APPT (Figure 2). It can be seen that discharge power mainly affects the degree of etching; while the original cotton fibre is flat and has a twisted structure, and the surface is smoother than surface of the APP treated cotton fibres. Specimens treated at discharge power of 130 W–140 W show a dense population of slits and grooves whereas continuous and deep cleavages are seen when discharge power is increased to 160 W or 170 W. Intermolecular hydrogen bonds are formed between parallel β-d-glucopyranose polymer chains in cellulose molecules after APPT. Discharge power of 160 W to 170 W releases enough energy to etch the fibre surface intensively. The clearly identifiable change in morphology of fibres leads to an enhanced surface-to-volume ratio which in turn results in higher water absorption [14,15].

Chemical composition of the fibre surface up to a depth of 5 nm was examined by XPS for carbon (C), oxygen (O) and hydrogen (H), main components of cotton fibre, but H was not examined because XPS cannot detect it. Proportions of C and O in untreated and treated samples are shown in Table 2. 

Oxygen to carbon ratio (O/C ratio) of untreated sample was found to be 0.6 and after APP treatment O content increased while C decreased. O/C ratio of specimen treated at different discharge power was 0.9. This substantive change in fibre composition indicates substantial oxidization of the substrate because of the use of oxygen plasma. When the O/C ratio is taken into consideration, the increase in discharge power gives similar O/C ratio which means than the oxidation is a steady effect in applying plasma for treating cotton fibre in this study. 

Deconvolution analysis of C–C (285.0 eV) and C–O (286.5 eV) with C1s was carried by using XPSPEAK software; results are as presented in Table 3. Proportion of C–C obviously declines significantly while that of C–O functionality (any functional group contains C–O bonding) increases after APP treatment at atmospheric pressure. Obviously C–C and C–H bonds on the surface of cotton fibre are broken because of attack of the reactive plasma species; oxygen atoms/radicals get combined with carbon radicals [21,22], leading to groups containing oxygen on the surface. The lower C–O content in 150 W and 170 W power clearly means higher power discharge does not enhance C–O. 

FTIR-ATR was used to examine functionality at 500 nm depth. Table 4 shows the peak area of 3270 cm^−1^ which corresponds to magnitude of C–OH stretching in alcohol while the peak area at 1313 cm^−1^ is corresponding to C–O stretching of ether.

FTIR results (Table 4) show that APPT generates functional groups such as C–OH and C–O, which changes functionality of the surface in terms of ability to absorb water since intensive hydrogen bonds are formed with water molecules. As Table 2 shows, these functional changes are better achieved at discharge power of 130 W to 150 W (0.3 L/min oxygen) whereas the phenomenon is less pronounced at higher discharge power of 170 W. A higher discharge power means release of more energy, which results in C–OH and C–O since active species become more concentrated. In case of discharge power of 170 W, intensity of active species is too high and C–O–C bonds between different units of cellulose are broken, causing the higher etching at the surface. Therefore, the quantity of C–O drops significantly. 

### 3.2. Effect of Oxygen Flow Rate

**Figure 3:**
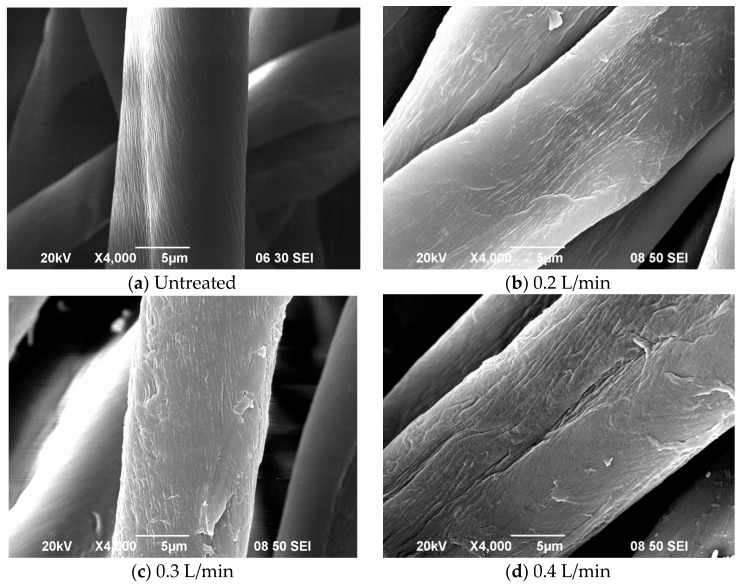

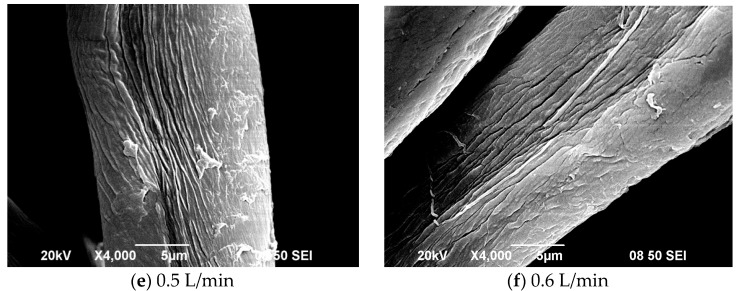
SEM images of (**a**) untreated cotton fibre (Ref. [21], with permission); APP treated cotton fibre with oxygen flow rate of (**b**) 0.2 L/min; (**c**) 0.3 L/min; (**d**) 0.4 L/min; (**e**) 0.5 L/min; and (**f**) 0.6 L/min (APP conditions: Discharge power = 170 W; jet-to-substrate distance = 3 mm and jet moving speed = 5 mm/s).

Figure 3 below shows the effect of oxygen flow rate on morphological changes on the fibre surface. 

As the oxygen flow rate increases from 0.2 L/min to 0.6 L/min, roughness of surface of treated fibre increases. Deep and continuous cracks formed along the fibre axis in crumbly layers constitute the most pronounced morphological change. The cracks are relatively finer and shallow until an oxygen flow rate of 0.4 L/min and when the flow rate is increased further, the cracks become deep and wide grooves.

Table 5 shows results of XPS analysis of C and O contents at 5 nm depth. As can be seen in Table 5, as the oxygen flow rate increases, quantity of C decreases while that of O content increases sharply. Thus, the O/C ratio increases significantly. The substantive increase in C–O present on the surface after APPT is attributed to the high degree of oxidation. However, etching causes only a small change in surface, as is evident from an O/C ratio of 5:6 per unit of cellulose. When the surface of the fabric is bombarded by active plasma species, C free radicals are formed in large quantities because the C–C bonds get broken and react with oxygen free radicals to form C–O bonding (Table 6). Absorbency rises significantly after APPT because of the higher O content which leads to formation of hydrogen bonds with water molecules [23,24,25]. 

A higher flow rate of oxygen, up to 0.6 L/min, however, reduces oxygen content as well as C–O (Figure 3; SEM images) as the slits on fibre surface are generated in larger numbers. This is attributable to etching caused by the high oxygen flow rate and discharge power. When the O/C ratio is taken into consideration, the increase in oxygen flow rate gives similar O/C ratio which means than the oxidation is a steady effect in applying plasma for treating cotton fibre in this study. 

Results of FTIR (Table 7) confirm that C–OH and C-O functional groups are present on the cotton fibre surface. Oxidation results in increased number of C–OH and C–O groups. C–C and C–H bonds that get broken form carbon radicals by reacting with oxygen and/or radicals/atoms. Until discharge power reaches 170 W, intensity of C–OH and C–O groups remains proportional to oxygen flow rate. However, XPS and FTIR show different trends in terms of C–O quantity which is because of the large difference in depth of the measurements 5 nm in case of XPS against 500 nm in FTIR. 

### 3.3. Effect of Jet Moving Speed

**Figure 4:**
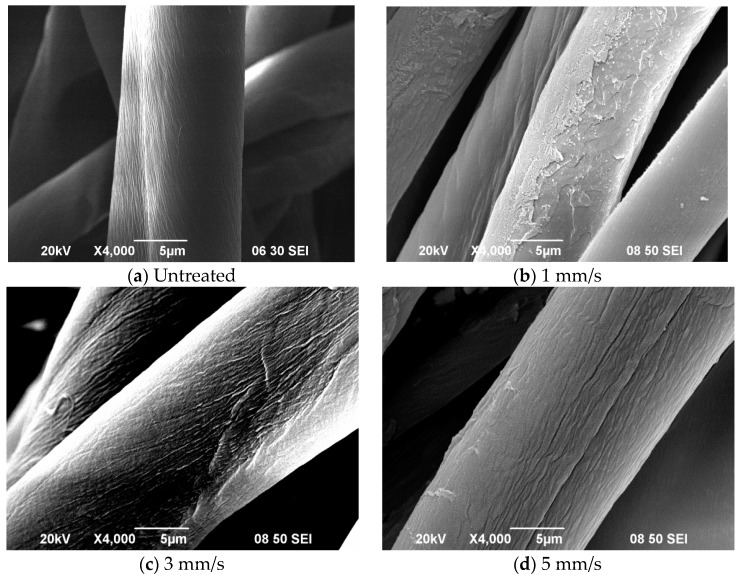

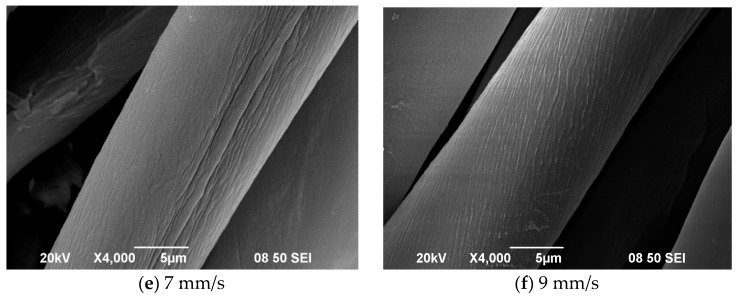
SEM images of (**a**) untreated cotton fibre, APP treated cotton fibre with jet moving speed of (**b**) 1 mm/s; (**c**) 3 mm/s; (**d**) 5 mm/s; (**e**) 7 mm/s (For Figure 4a–e, Ref. [21], with permission) and (**f**) 9 mm/s (APP conditions: Discharge power = 150 W; oxygen flow rate = 0.4 L/min and jet-to-substrate distance = 3 mm).

The magnitude of morphological changes in terms of roughness of surface of the substrate depends upon the duration of the APPT, defined as the time period for which the fabric remains under the jet. This is clear from Figure 4, which shows SEM images of cotton fibres treated at speeds of 1 mm/s, 3 mm/s, 5 mm/s, 7 mm/s and 9 mm/s. As the duration increases, i.e., jet moving speed is reduced from 9 mm/s to 1 mm/s, the number of voids and cracks generated increases. At a jet speed of 1 mm/s isolated spots can be seen on the surface and continuous cracks can still be observed. Shorter APPT durations 3 mm/s to 9 mm/s) lead to formation of long fine lines and non-directional isotropic patterns of etching can be observed. On the other hand slower nozzle movement (longer treatment duration) means continuous bombardment with active plasma species which causes tiny spots to appear because of etching effect. Some of the substances also get re-deposited sometimes.

Duration of APPT also affects chemical composition at the surface (Table 8). Oxygen plasma reduces the carbon content and increases oxygen. Table 9 shows results of deconvolution of C1s spectra in terms of proportions of C–C and C–O up to depth of 5 nm. While C–C declines significantly, there is a notable increase in C–O after the APPT. Absorbency of the fabric is vastly enhanced because of the C–O group which attracts water molecules [26,27]. 

As the jet moving speed increases from 1 mm/s to 5 mm/s proportion of O decreases but when speed increases further from 5 mm/s to 9 mm/s there is an increase in it. On the other hand, C–O increases consistently with increase of jet moving speed, from 1 mm/s to 9 mm/s. The etching effect also decreases steadily as jet moving speed rises from 1 mm/s to 9 mm/s (Figure 4). More of cracks and voids are generated on the surface as jet speed decreases from 9 mm/s to 1 mm/s because a slower speed implies accumulation of more active plasma species. Surface oxidation has small fluctuations (carbon and oxygen content) when jet speed changes from 1 mm/s to 5 mm/s and then to 9 mm/s. It seems that C–O groups get etching away at 1 mm/s jet moving speed. When the O/C ratio is taken into consideration, the increase in jet moving speed oxygen flow rate give similar O/C ratio which means than the oxidation is a steady effect in applying plasma for treating cotton fibre in this study. 

Functional groups C–OH and C–O on the fibre surface increase after the APPT (Table 10). When active plasma species hit the surface, carbon radicals are formed because of breaking of C–C and C–H bonds. These carbon radicals react with oxygen atoms and radicals in plasma or hydrogen in air. Density of C–OH increases substantially when jet speed increases from 1 mm/s to 9 mm/s (Table 10). Jet speed affects the quantum of C–O which is different in XPS and FTIR because of the difference in depth of measurements. 

### 3.4. Effect of Jet-to-Substrate Distance

**Figure 5:**
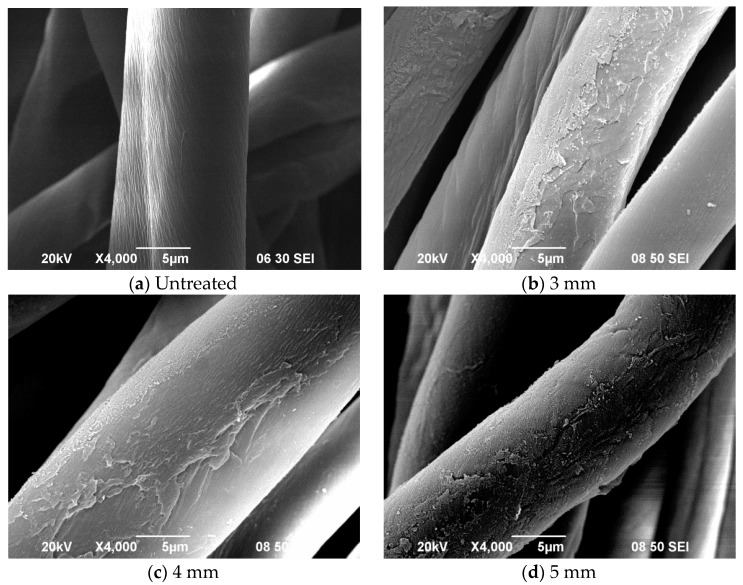

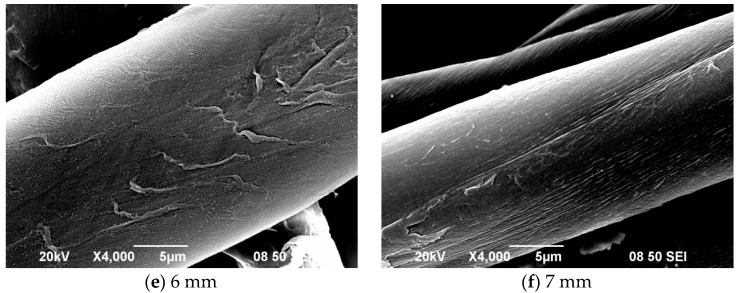
SEM images of (**a**) untreated cotton fibre, APP treated cotton fibre with jet-to-substrate distance of (**b**) 3 mm (For Figure 5a,b, Ref. [21], with permission), (**c**) 4 mm, (**d**) 5 mm, (**e**) 6 mm and (**f**) 7 mm (APP conditions: Discharge power = 150 W; oxygen flow rate = 0.4 L/min and jet moving speed = 1 mm/s).

Figure 5 shows how jet-to-substrate distance affects etching, which generates cleavages appear on APP plasma treated fibres. Figure 5b,c show several spots of cleavages on fabrics treated with jet-to-substrate distance of 3 mm and 4 mm respectively. 

Evidently, spot formation is high when jet-to-substrate distance is small, such as 3 mm or 4 mm, indicating that the smaller the distance is the higher is the spot density. Fine slits (Figure 5f) are attributable to some plasma etchants having less energy which have shorter lifespans, besides the fact that some species lose energy because of collisions. However, excessively small distance between the nozzle and the fabric can result in glass transition leading to some change in bulk property because of high temperature. 

Results of XPS analysis for examining the effect of jet-to-substrate distance on composition of cotton at the fabric surface (Table 11) shows that proportion of O increases dramatically when the distance is 3 mm and 5 mm. However, when the distance is increased from 5 mm to 7 mm, O percentage decreases. One reason for this is that when the distance increases beyond a threshold (i.e., 5 mm), active plasma species are unable to preserve energy required for forming new polar groups. When the O/C ratio is taken into consideration, the increase in jet-to-substrate distance gives similar O/C ratio which means than the oxidation is a steady effect in applying plasma for treating cotton fibre in this study. 

Deconvolution analysis was used for derivation of C–C (285.0 eV) and C–O (286.5 eV) with C1s (Table 12) and C–C percentage was found to have declined remarkably while C–O increased significantly after oxygen APPT. C–O groups were generated on polymer surface quite densely as carbon radicals and oxygen species reacted and combined with each other.

Table 11 and Table 12 show the trends of proportion of oxygen and C–O respectively; when jet-to-substrate distance is 5 mm, O content is maximum but C–O is minimum, within a set range. Oxygen containing groups –OH, –COOH, –COH, etc. result in high oxygen content. At jet-to-substrate distance of 7 mm, though O content declined, C–O increased. Oxygen containing groups besides –C–O were removed due to etching at jet-to-substrate distance of 5 mm. 

The functional groups as C–O were generated on the fibre surface during and/or after oxygen APPT is obvious from the significant increase in the peak area of C–O. Bombardment by energy carrying active plasma species breaks the C–C and C–H bonds and the newly formed carbon radicals then react with oxygen or hydrogen species. The C–OH density increases significantly as jet-to-surface distance increases from 3 mm to 5 mm, though there is a slight decrease when the distance further increases to 7 mm (Table 13). 

At jet-to-substrate distance of 5 mm or larger, etching is relatively mild, as shown by SEM images (Figure 5), than 5 mm because oxidation results in gentle etching. Again, results of XPS and FTIR vary because of the difference in depth of measurement. 

## 4. Conclusions

The impact of different operational parameters of APPT, discharge power, oxygen flow rate, jet-to-substrate distance and jet moving speed, on surface properties of cotton fibre, is examined. Surface properties were characterised by SEM, XPS and FTIR-ATR. It was found that the effects vary with different combinations of the parameters. Generally speaking, under the influence of APPT, the cotton surface was roughened as revealed by SEM images. On the other hand, oxygen content on the surface of APP treated cotton was increased significantly, which would enhance the wettability of the cotton fibre. In this study, we revealed that the operational parameters would physical and chemically after the surface characteristics of the cotton fibre. Physically, cracks and grooves were noted in the cotton fibre surface after APPT. Chemically, the oxygen content in the cotton fibre surface was increased after APPT. When the O/C ratio is taken into consideration, the surface oxidation was a steady effect in applying APPT for treating cotton fibre in this study. Therefore, the operational parameters need to be chosen carefully when subjecting fabric to atmospheric pressure plasma treatment with oxygen in order to achieve desired functional effect. 

## Figures and Tables

**Table 1:** Operational parameters studied used in the APPT.

Operational Parameter	Unit	Range
Discharge power	W	130–170
Oxygen flow rate	L/min	0.2–0.6
Jet moving speed	mm/s	1–9
Jet-to-substrate distance	mm	3–7

**Table 2:** XPS analysis of cotton samples (effect of discharge power).

Fixed Parameters	Discharge Power (W)	C%	O%	O/C Ratio
Oxygen flow rate = 0.3 L/min; Jet moving speed = 5 mm/s; and Jet-to-substrate distance = 3 mm	Control	64.1	35.9	0.6
	130	53.7	46.3	0.9
	150	51.5	48.5	0.9
	170	53.6	46.4	0.9

C and O represent carbon and oxygen correspondingly.

**Table 3:** XPS deconvolution analysis of relative chemical bonds of cotton samples (effect of discharge power).

Fixed Parameters	Discharge Power (W)	C–C%	C–O%
Oxygen flow rate = 0.3 L/min; Jet moving speed = 5 mm/s; and Jet-to-substrate distance = 3 mm	Control	47.8	52.2
	130	11.0	89.0
	150	16.0	84.0
	170	17.0	83.0

**Table 4:** FTIR analysis of relative intensity of chemical bonds of cotton samples with variation of discharge power.

Fixed Parameters	Discharge Power (W)	C–OH	C–O
Oxygen flow rate = 0.3 L/min; Jet moving speed = 5 mm/s; and Jet-to-substrate distance = 3 mm	Control	4.1	2.3
	130	7.2	3.8
	150	7.3	4.0
	170	5.6	3.0

**Table 5:** XPS analysis of cotton samples (effect of oxygen flow rate).

Fixed Parameters	Oxygen Flow Rate (L/min)	C%	O%	O/C Ratio
Discharge power = 170 W; Jet moving speed = 5 mm/s; and Jet-to-substrate distance = 3 mm	Control	64.1	35.9	0.6
	0.2	53.1	46.9	0.9
	0.4	53.2	46.8	0.9
	0.6	54.2	45.8	0.8

**Table 6:** XPS deconvolution analysis of relative chemical bonds of cotton samples (effect of oxygen flow rate).

Fixed Parameters	Oxygen Flow Rate (L/min)	C–C%	C–O%
Discharge power = 170 W; Jet moving speed = 5 mm/s; and Jet-to-substrate distance = 3 mm	Control	47.8	52.2
	0.2	11.7	88.3
	0.4	19.7	80.3
	0.6	18.2	81.8

**Table 7:** FTIR analysis of relative intensity of chemical bonds of cotton samples with variation of oxygen flow rate.

Fixed Parameters	Oxygen Flow Rate (L/min)	C–OH	C–O
Discharge power = 170 W; Jet moving speed = 5 mm/s; and Jet-to-substrate distance = 3 mm	Control	4.12	2.30
	0.2	5.61	2.16
	0.4	6.41	3.35
	0.6	7.27	3.75

**Table 8:** XPS analysis of cotton samples (effect of jet moving speed).

Fixed Parameters	Jet Moving Speed (mm/s)	C%	O%	O/C Ratio
Discharge power = 150 W; Oxygen flow rate = 0.4 L/min; and Jet-to-substrate distance = 3 mm	Control	64.1	35.9	0.6
	1	55.1	44.9	0.8
	5	55.8	44.2	0.8
	9	55.4	44.6	0.8

**Table 9:** XPS deconvolution analysis of relative chemical bonds of cotton samples (effect of jet moving speed).

Fixed Parameters	Jet Moving Speed (mm/s)	C–C%	C–O%
Discharge power = 150 W; Oxygen flow rate = 0.4 L/min; and Jet-to-substrate distance = 3 mm	Control	47.8	52.2
	1	30.9	69.1
	5	23.8	76.2
	9	19.8	80.2

**Table 10:** FTIR analysis of relative intensity of chemical bonds of cotton samples with variation of jet moving speed (Ref. [21], with permission).

Fixed Parameters	Jet Moving Speed (mm/s)	C–OH	C–O
Discharge power = 150 W; Oxygen flow rate = 0.4 L/min; and Jet-to-substrate distance = 3 mm	Control	4.1	2.3
	1	9.7	4.8
	5	8.3	4.3
	9	7.0	4.2

**Table 11:** XPS analysis of cotton samples (effect of jet-to-substrate distance).

Fixed Parameters	Jet-to-Substrate Distance (mm)	C%	O%	O/C Ratio
Discharge power = 150 W; Oxygen flow rate = 0.4 L/min; and Jet moving speed = 1 mm/s	Control	64.1	35.9	0.6
	3	55.3	44.7	0.8
	5	55.1	44.9	0.8
	7	55.7	44.3	0.8

**Table 12:** XPS deconvolution analysis of relative chemical bonds of cotton samples (effect of jet-to-substrate distance).

Fixed Parameters	Jet-to-Substrate Distance (mm)	C–C%	C–O%
Discharge power = 150 W; Oxygen flow rate = 0.4 L/min; and Jet moving speed = 1 mm/s	Control	47.8	52.2
	3	23.8	76.2
	5	25.7	74.3
	7	20.5	79.5

**Table 13:** FTIR analysis of relative intensity of chemical bonds of cotton samples with variation of jet-to-substrate distance.

Fixed Parameters	Jet-to-Substrate Distance (mm)	C–OH	C–O
Discharge power = 150 W; Oxygen flow rate = 0.4 L/min; and Jet moving speed = 1 mm/s	Control	4.1	2.3
	3	8.3	4.3
	5	10.7	5.2
	7	10.2	4.9
